# Prostaglandin E2 and Receptors: Insight Into Tumorigenesis, Tumor Progression, and Treatment of Hepatocellular Carcinoma

**DOI:** 10.3389/fcell.2022.834859

**Published:** 2022-03-10

**Authors:** Chao Chen, Jun Guan, Xinyu Gu, Qingfei Chu, Haihong Zhu

**Affiliations:** State Key Laboratory for Diagnosis and Treatment of Infectious Diseases, National Clinical Research Center for Infectious Diseases, National Medical Center for Infectious Diseases, Collaborative Innovation Center for Diagnosis and Treatment of Infectious Diseases, The First Affiliated Hospital, Zhejiang University School of Medicine, Hangzhou, China

**Keywords:** prostaglandin E2, EP receptor, hepatocellular carcinoma, tumorigenesis, tumor progression, tumor treatment

## Abstract

Hepatocellular carcinoma (HCC) is a common primary liver cancer with ∼750,000 annual incidence rates globally. PGE2, usually known as a pro-inflammatory cytokine, is over-expressed in various human malignancies including HCC. PGE2 binds to EP receptors in HCC cells to influence tumorigenesis or enhance tumor progression through multiple pathways such as EP1-PKC-MAPK, EP2-PKA-GSK3β, and EP4-PKA-CREB. In the progression of hepatocellular carcinoma, PGE2 can promote the proliferation and migration of liver cancer cells by affecting hepatocytes directly and the tumor microenvironment (TME) through ERK/COX-2/PGE2 signal pathway in hepatic stellate cells (HSC). For the treatment of hepatocellular carcinoma, there are drugs such as T7 peptide and EP1 antagonist ONO-8711 targeting Cox-2/PGE2 axis to inhibit tumor progression. In conclusion, PGE2 has been shown to be a traditional target with pleiotropic effects in tumorigenesis and progression of HCC that could be used to develop a new potential clinical impact. For the treatment study focusing on the COX-PGE2 axis, the exclusive usage of non-steroidal anti-inflammatory agents (NSAIDs) or COX-2-inhibitors may be replaced by a combination of selective EP antagonists and traditional anti-tumoral drugs to alleviate severe side effects and achieve better outcomes.

## Introduction

Hepatocellular carcinoma (HCC) accounts for 90% of primary liver cancers with an annual incidence rate of ∼750,000, ranking as the fifth most common malignant tumor worldwide in men and seventh in women (Ferlay et al., 2010; Hepatocellular carcinoma, 2016). Due to complications such as gastrointestinal bleeding, liver failure, cachexia, and tumor rupture, HCC patients always have a short survival period (Leng et al., 2003). Surgical resection is considered as the best treatment method, but advanced-stage HCC, which is unqualified for surgery, remains a challenge. Compared to the natural history of advanced-stage liver cancer, systemic therapies based on tyrosine kinases (TKIs) and immune checkpoint inhibitors (ICIs) have improved patients’ outcomes, and prolonged life expectancy significantly in recent years (Yang et al., 2020). But there is still great demand for the new drugs due to the variability of hepatocellular carcinoma. Anti-inflammation is an important strategy for treatment as inflammation is a key progress in the tumorigenesis and progression of tumor. PGE2, usually known as a pro-inflammatory cytokine, is over-expressed in various human malignancies (Wang and Dubois, 2010). Inhibiting PGE2 synthesis has been an important anti-inflammatory strategy for more than 100 years (Vane and Botting, 2003). Non-steroidal anti-inflammatory agents (NSAIDs), such as aspirin, mainly take effect by inhibiting cyclooxygenase (COX) while prostaglandin E2 (PGE2) is the primary metabolic product in inflammation. Epidemiological observation of several common cancers shows a positive correlation between aspirin and lower death rate (Rothwell et al., 2011; Lin et al., 2018; Ma and Brusselaers, 2018; Cho et al., 2021), indicating that reduction of PGE2 may prevent solid-organ cancers such as HCC (Wu, 2006).

## Biosynthesis of Prostaglandin E2

**FIGURE 1:**
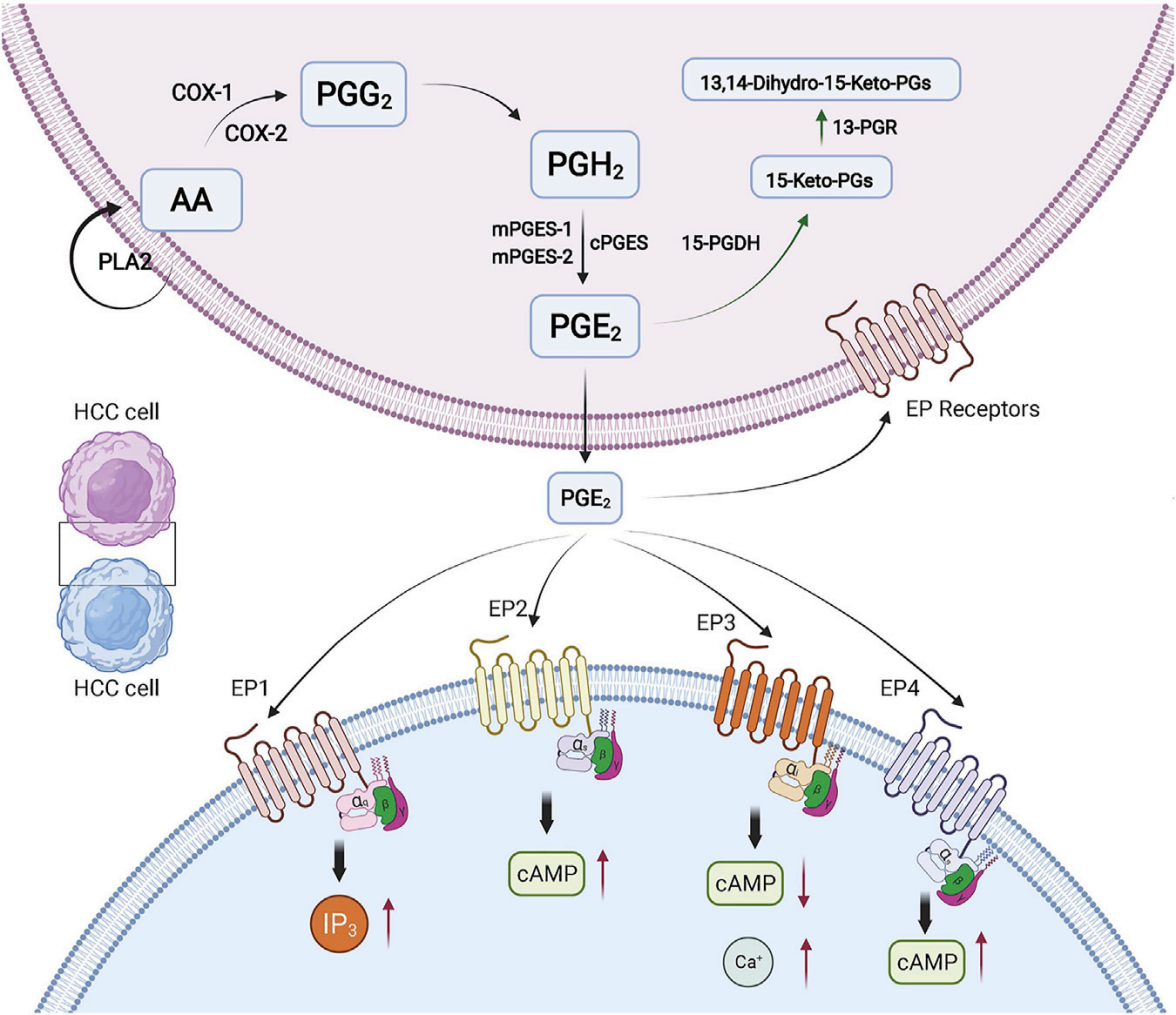
PGE2 in HCC. The biosynthesis and function of PGE2 in HCC is shown. The red arrows indicate the changed expression of molecules. The green arrows indicate the degradation pathway of PGE2. Created with BioRender.com. 15-PGDH, 15-hydroxyprostaglandin dehydrogenase; 13-PGR, 15-keto-prostaglandin-(13)-reductase.

Prostaglandins (PGs) belong to the eicosanoid family and are synthesized by almost all kinds of cells in the human body, in which PGE2 is the most abundant (Park et al., 2006). When cells are stimulated by growth factors, cytokines, inflammatory mediators, or various cancer-promoting factors, and the expression of PGE2 can be significantly increased. The synthesis of PGE2 is derived from a polyunsaturated fatty acid Arachidonic Acid (AA), which is released from cell membrane by phospholipases A2 (PLA2). Then AA is oxidized to the prostaglandin G2 (PGG2) and turns into unstable intermediate prostaglandin H2 (PGH2) under the action of COX. PGH2 will be converted into PGE2 rapidly by the help of three distinct synthases: microsomal prostaglandin E2 synthase-1 (mPGES-1), microsomal prostaglandin E2 synthase-2 (mPGES-2), and cytosolic prostaglandin E2 synthase (cPGES). COX is the key enzyme in the AA metabolism pathway. COX has two kinds of isoforms, namely COX-1 and COX-2, which play different roles in physiological or pathological conditions. Generally, COX-1 is continuously expressed in most tissues, controlling physiological functions such as regulating vascular homeostasis or cellular responses to hormone stimulation. COX-2 is mainly expressed after growth factor action or inflammation stimulation, which often leads to the increasing synthesis of PGE2 in tumor tissue or inflammation tissue (Tanabe and Tohnai, 2002). Evidence suggests that there is preferential functional coupling in the interactions between COX and PGES, while mPGES-1 tends to couple with COX-2 and cPGES preferentially couples with COX-1 (Muthalif et al., 2001; Ouellet et al., 2002; Murakami et al., 2003). The key enzymes of the catabolic process of PGE2 include 15-ketoprostaglandin-13-reductase (13-PGR) and 15-hydroxy-prostaglandin dehydrogenase (15-PGDH) (Tai, 2011) (Figure 1).

## PGE2 Receptors in Hepatocellular Carcinoma

**FIGURE 2:**
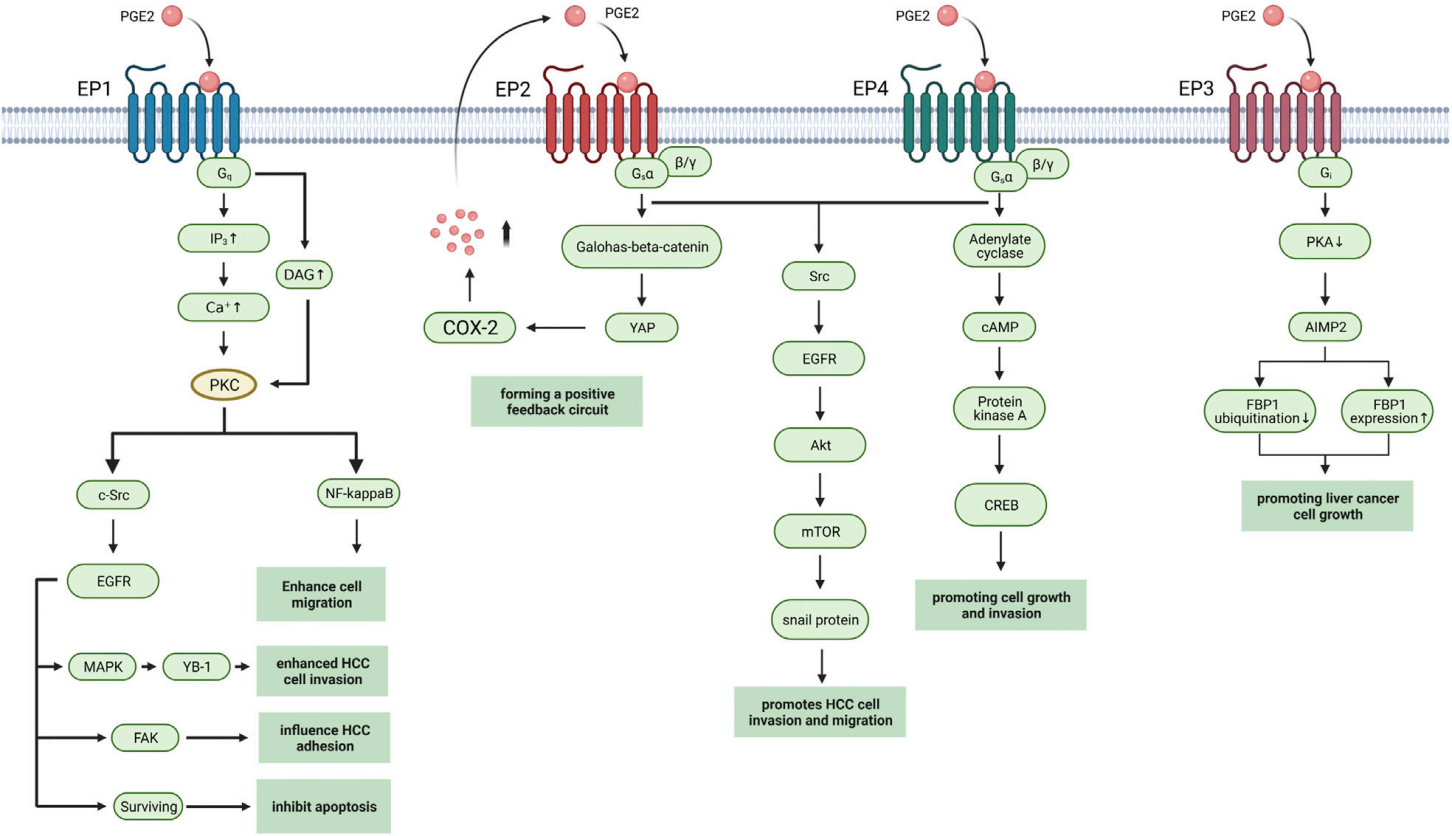
EP receptors in HCC. The EP receptors with downstream pathways in HCC are shown. Created with BioRender.com. YB-1, Y box-binding protein 1; FAK, Focal adhesion kinase; YAP, Yes-associated protein; AIMP2, aminoacyl-tRNA synthetase interacting multifunctional protein 2, also known as JTV1; FBP1, FUSE-binding protein 1.

PGE2 execute physiological or pathological activities by binding to seven transmembrane G-protein coupled receptors (GPCRs), which are known as prostaglandin E (EP) receptors. EP receptors can divide into four isoforms: EP1-4. Heterotrimeric G proteins couple with EP receptors containing stimulatory subunit (GαS) or inhibitory subunit (Gαi) which can regulate the levels of cytoplasmic cyclic AMP (cAMP), Ca2+, and inositol phosphate to activate different downstream signaling pathways (Figure 2). Normally PGE2 binds to the EP receptors in immunocyte, playing a pro-inflammatory or anti-inflammatory role due to the type of EP receptor and immunocyte (An et al., 1993; Reinold et al., 2005; Sugimoto and Narumiya, 2007). But in hepatocellular carcinoma cell line and tumor, the activation of EP receptors in HCC cell influence tumorigenesis, and tumor progression through multiple pathways (Table 1).

**TABLE 1:** The EP receptor in the HCC.

EP receptor	Target	Signal	Description	References
EP1	Hepatocyte	EP1/PKC/c-Src/EGFR/MAPK/YB-1	enhanced HCC cell invasion	Han et al., (2006), Zhang et al., (2014a)
	Hepatocyte	EP1/PKC/c-Src/EGFR/FAK	influence HCC adhesion	Bai et al. (2013)
	Hepatocyte	EP1/PKC/c-Src/EGFR/surviving	inhibitor of apoptosis protein family	Bai et al. (2010)
	Hepatocyte	EP1/PKC/NF-kappaB/FoxC2/β1-integrin	cell migration	Bai et al. (2014)
	Hepatocyte	EP1/HIF-1α	Activate mTOR signaling pathway	Ji et al. (2010)
EP2	Hepatocyte	EP2/Src/EGFR/Akt/mTOR/snail	promotes HCC cell invasion and migration	Cheng et al. (2014)
	Hepatocyte	COX-2-PGE2-EP2-Galphas-beta-catenin-YAP-COX-2	forming a positive feedback circuit	Xu et al. (2018)
EP3	Hepatocyte	EP3/PKA/JTV1/FBP1	inhibiting the ubiquitination of FBP1 and increasing FBP1 protein expression, promoting liver cancer cell growth	Ma et al. (2013)
EP4	Hepatocyte	EP4-PKA-CREB	Induce proto-oncogene c-Myc	Xia et al. (2014)
	Hepatocyte	inducing overexpression of anti-apoptotic protein Bcl-xL	hepatocyte protective effects	Ushio et al. (2004)
	Hepatic stellate cells	COX2-PGE2-EP4	accumulate immunosuppressive cell as Th17 cells, regulate T cells and myeloid-derived suppressor cells (MDSCs)	(Li et al., 2017), (Xu et al., 2016)

EP1 receptor usually couples with the Gαq protein subunit, which increases the level of intracellular Ca2+ and activate protein kinase C (PKC), thus inducing gene transcription through regulating the MAPK, nuclear factor-kappaB (NFκB), and nuclear factor of activated T cells (NFAT) pathways (Sugimoto and Narumiya, 2007). Clinical studies show that high EP1 receptor expression in tumor is associated with poor overall survival (OS) and poor differentiation of tumor (Yang et al., 2016). In HCC cell line, researchers found that EP1/PKC/c-Src signaling pathway can modulate the activation of epidermal growth factor receptor (EGFR) (Han et al., 2006). Then activated EGFR induced the phosphorylation of p44/42 MAPK, which in turn up-regulated Y box-binding protein 1 (YB-1) to promote cell invasion (Zhang et al., 2014a). The other targets of EGFR include focal adhesion kinase (FAK) which influences cell adhesion and survival by inhibiting apoptosis progress (Bai et al., 2010; Bai et al., 2014) Activation of PKC also induced β1-integrin over-expression and cell migration via NF-kappa B/FoxC2 signaling pathway through EP1 receptor to up-regulate β1-integrin expression and cell migration (Bai et al., 2014). The mTOR signaling pathway is also activated while EP1 agonist up-regulates HIF-1 alpha in HCC cell line (Ji et al., 2010).

EP2 receptor mainly couples with the Gαq protein subunit that activates adenylate cyclase to generate cAMP. The increasing cAMP in plasma activates the protein kinase A (PKA) to mediate glycogen synthase kinase 3β (GSK3β)-β catenin pathways (Narumiya et al., 1999).

Researchers found that activation of EP2 receptor enhances Hep3B human hepatocellular carcinoma cell line proliferation while antagonism of EP2 blocked growth and invasion induced by PGE2 (Guo et al., 2011; Zang et al., 2017). And activation of EP2 upregulated snail protein through Src-EGFR-Akt-mTOR pathway to enhance tumor cell migration (Cheng et al., 2014). In HCC cell line, EP2 also mediated the transcriptional induction of Yes-associated protein (YAP) via Wnt/β-Catein pathway while YAP can interact with the COX-2 promoter to increase COX-2 expression, thus forming a positive feedback circuit (Xu et al., 2018).

EP3 receptor is a G-protein-coupled receptor with multiple C-terminal tails due to alternative mRNA splicing (Kotani et al., 1997). Cell experiments show that EP3 can down-regulate the expression of aminoacyl-tRNA synthetase interacting multifunctional protein 2 (AIMP2) to inhibit the ubiquitination of FUSE-binding protein 1(FBP1) and increase FBP1 protein expression through Gs protein and PKA pathway, thus promoting cell growth (Ma et al., 2013). But further study of EP3 in HCC has produced unclear results.

Activation of EP4 also upregulates snail protein via EGFR to promote migration in hepatoma cells (Zhang et al., 2014b). Proto-oncogene c-Myc is induced through EP4-PKA-CREB signaling pathway (Xia et al., 2014). EP4-receptor agonist (PGE2R-A) shows hepatocyte protective effects in HepG2 HCC cells by inducing overexpression of anti-apoptotic protein Bcl-xL (Ushio et al., 2004). The PGE2 synthesized by Hepatic stellate cells (HSCs) accumulates immunosuppressive cells, such as Th17 cells, and regulates T cells and myeloid-derived suppressor cells (MDSCs) via EP4 receptor (Xu et al., 2016; Li et al., 2017).

## Overexpression of PGE2 in Hepatocellular Carcinoma

Clinical research has discovered that PGE2 in the peripheral blood of patients with hepatocellular carcinoma has increased significantly. A further study found that COX-2 expression in HCC tissues is higher than in adjacent tissues and in normal liver tissues (Cervello and Montalto, 2006). As for mPGES-1, which synthesize PGE2 directly by binding to Cox-2, this also increased in HCC tissue (Zang et al., 2013), indicating that increased PGE2 in HCC patients is synthesized by HCC tissues. The increased expression of PGE2-related synthase in HCC is closely related to various carcinogenic factors.

Viral hepatitis is one of the main causes of hepatocellular carcinoma. HBV and HCV can promote the expression of PGE2 in liver cancer tissues in different ways. HBx protein is one of the HBV virus proteins that has a wide range of transactivation functions and plays an important role in cell proliferation, apoptosis, and genetic stability of liver cells.

Transfected with HBx, Hep3B hepatocellular carcinoma cells overexpress COX-2, and generates more PGE2 (Cheng et al., 2004). In HBx-positive cells of chronic hepatitis B patients, HBx protein reduces the function of DNA methyltransferase, which in turn increases methylation site in the CpG dinucleotide of the COX-2 promoter. Methylation increases binding affinity of the COX-2 promoter to increase COX-2 expression in hepatocytes, which ultimately leads to higher level of circulating PGE2 in peripheral blood (Yue et al., 2011). HBx protein is also positively correlated with mPGES-1 expression in cancer tissues of HBV-related HCC patients. The researchers believe that HBx may also promote the expression of PGE2 by increasing the level of early growth response 1 (EGR1) that binds to the transcription site of the mPGES-1 promoter (Liu et al., 2014). HCV can increase PGE2 synthesis by non-structural protein NS3 which can enhance the activity of the COX-2 gene promoter in HepG2 cells through a transcription factor NF-κB-dependent pathway (Lu et al., 2008).

In addition to viral hepatitis, there are a variety of factors that induce the production of prostaglandin-related synthase in non-viral non-cirrhotic HCC. Obesity is one of the most important pathogenic factors. The intestinal microflora of obese people secretes lipoprotein phosphate (LTA) and intestinal microbial metabolite deoxycholic acid (DCA), which synergistically enhances hepatic stellate cells (HSC) aging-related secreted phenotype (SASP), through Toll -Like receptor 2 (TLR2), up-regulating the expression of COX-2, thereby promoting the synthesis of PGE2 (Loo et al., 2017). The LPS secreted by abnormal intestinal flora can interact with TLR4 in HCC to induce PGE2 expression through COX-2/PGE2/STAT3 positive feedback loop.

At the same time, the LPS secreted by the abnormal intestinal flora can also activate the COX-2/PGE2/STAT3 positive feedback loop through the functional expression of TLR4 on HCC cells (Lin et al., 2016).

Overexpression of PGE2 is also related to the severity and prognosis of HCC patients. Correlation analysis found that the patients with higher circulating PGE2 levels have larger tumor size and shorter overall survival (Tai et al., 2019; Pelizzaro et al., 2021). Patients with portal vein thrombosis, non-enveloped, and advanced stage disease according to Barcelona Clinic Liver Cancer (BCLC) have higher PGE2 levels in their cancer tissues, suggesting that PGE2 may be related to the aggressiveness of liver cancer (Zang et al., 2017). There is also a significant positive correlation between the level of mPGES-1 and the clinical liver cancer stage of BCLC (Zang et al., 2013), while patients with higher COX-2 expression are more likely to suffer from lymphatic infiltration and distant metastasis of HCC (Tai et al., 2019).

## Two Opinions About Effect of PGE2 on Tumorigenesis of Hepatocellular Carcinoma

The tumorigenesis of hepatocellular carcinoma is a complex multi-step process involving persistent inflammatory damage, hepatocyte necrosis, regeneration, and fibrosis deposition. Nearly 70–90% of hepatocellular carcinom**a** cases have a history of chronic liver disease or cirrhosis (El–Serag and Rudolph, 2007), while PGE2 shows high levels in chronic hepatitis patient and even higher levels in patients with cirrhosis, suggesting that PGE2 may promote the tumorigenesis of hepatocellular carcinoma. But there is also some evidence which shows the tumorigenesis of hepatocellular carcinoma is unrelated to PGE2.

### PGE2 Promote the Tumorigenesis of Hepatocellular Carcinoma

In clinical practice, non-steroidal anti-inflammatory drugs such as aspirin are widely used. The epidemiological studies on HCC have shown that the long-term use of small doses of aspirin can significantly reduce the risk of HCC. PGE2 is one of the most abundant COX-dependent prostaglandins in acute and chronic inflammation, indicating that PGE2 might be the major factor influencing the occurrence of HCC treated with long-term use of aspirin.

In animal experiments, the use of different doses of selective COX-2 inhibitors can significantly reduce the level of serum biochemical parameters of HCC induced by nitrosamine diethyl nitrosamine (DEN) in rats. Stained sections of rat liver tissues treated with high-dose COX-2 inhibitors are almost like normal tissues (Afzal et al., 2019). Deletion of the EP2 receptor gene leads to a reduction in the number and size of intestinal polyps in human familial adenomatous polyposis model mice (Sonoshita et al., 2001).

At the genetic level, the occurrence of hepatocellular carcinoma is not only related to epigenetic modifications, but also the result of cumulative changes in the genome of the hepatocyte. When changes in normal liver tissue affect the expression of oncogenes and tumor suppressor genes, it can further evolve the abnormal liver cell monoclonal population into HCC. Studies have shown that PGE2 can significantly up-regulate C-myc expression at both mRNA and protein levels, while knocking down C-myc can block PGE2-induced HCC cell growth and human hepatoma cell line Huh7 invasive ability; this process may be achieved through the EP4/GS/AC/cAMP/PKA/CREB signaling pathway (Xia et al., 2014).

### PGE2 Is Unrelated to the Tumorigenesis of Hepatocellular Carcinoma

But there is also some evidence to disprove the assumption that PGE2 promotes the tumorigenesis of hepatocellular carcinoma. Although long-term low-dose aspirin can reduce the incidence of HCC, the study of other NSAIDs such as ibuprofen found that its application has no significant relationship with the incidence of HCC (Petrick et al., 2015). In addition, aspirin inhibits the effects of COX-1 and COX-2 at the same time, but in the chronic hepatitis process that is closely related to cancer, it is generally believed that COX-2 plays a major role. However, no studies have reported that selective COX- 2 inhibitors are statistically related to HCC risk. These all suggest that the mechanism by which aspirin reduces the risk of HCC may be non-COX-dependent. The level of COX does not affect the pathogenesis of HCC. In the latest prospective study, researchers saved urine samples of 18,244 people based on prostaglandin E2 metabolite (PGE-M), a stable final metabolite, after catabolism of PGE2 mainly excreted in urine, and which represents the level of PGE2 synthesis in the human body over a period of time. After 28 years of follow-up, the number of liver cancer cases was counted, and the PGE-M level of corresponding urine was detected. There is no significant evidence to show the association between urine PGE-M level and the risk of HCC (Yuan et al., 2019). In animal model, studies on COX-2 transgenic mice with chemical carcinogens induced by diethylnitrosamine (DEN) have shown that overexpression of COX-2 is beneficial to the growth of pre-tumor tumors, but it will not affect their transformation into malignant tumors. (Llorente Izquierdo et al., 2011).

In general, PGE2 has shown the ability to promote the transformation of liver cells into cancer cells *in vitro* and vivo, but further clinical studies suggested that PGE2 may not play a significant role in the tumorigenesis of liver cancer. The discrepancies between two opinions are due to the unappropriated indicator PGE-M in clinical study, which reflects the PGE2 catabolism of multiple organs instead of just the liver. And mice model with chemical carcinogens induced by DEN is also not a qualified model to reflect PGE2 effect on tumorigenesis of liver cancer, as PGE2 is known as an inflammatory mediator.

## PGE2 Enhance Hepatocellular Carcinoma Progress

Evidence shows that PGE2 can promote HCC progression through autocrine and paracrine mechanisms by multiple ways (Table 2). *In vitro*, the downregulation of COX-2 expression can significantly inhibit HCC cell proliferation and colony formation, the downregulation of cell cycle-related protein cyclin D1, and lead to cell cycle arrest *in vitro* (Lv et al., 2019). In the mouse xenograft model, mPGES-1 overexpressed liver cancer cells form tumors faster and larger in nude mice, knockdown of mPGES-1 can delay tumor development and reduce tumor size (Lu et al., 2012), while research shows that the expression level of mPGES-1 in non-cancerous liver tissues can be used as a statistically important independent predictor of early recurrence after HCC (Nonaka et al., 2010).

**TABLE 2:** PGE2 enhance Hepatocellular Carcinoma metastasis and invasion through multiple recetports.

Model	Target		Specific role mechanism	References
Cell line	COX-2	Down regulate	inhibit cell proliferation and colony formation, down-regulation of cell cycle-related protein cyclinD1	Xu et al. (2018)
xenograft model mouse	mPGES-1	Up regulate	liver cancer cells form tumors faster and larger in nude mice	Kotani et al. (1997)
		Down regulate	knockdown of mPGES-1 can delay tumor development and reduce tumor size	
Cell line	EP1	activate	up-regulate β1-integrin expression and cell migration through PKC/NF-kappaB signaling pathway	Ma et al. (2013)
	EP1	activate	regulate FAK phosphorylation through PKC/c-Src and EGFR signaling pathways to enhance HCC adhesion and migration	Zhang et al. (2014b)
	EP2	activate	EP2-Src-EGFR-Akt-mTOR pathway to promoting the invasion and metastasis of liver cancer	Xia et al. (2014)
Tumor microenvironment	Mannan-binding lectin		directly interact with hepatic stellate cells (HSC) by inhibiting extracellular signal-regulated kinase (ERK)/COX-2/PGE2 signaling pathway and inhibit HCC-induced HSC activation	Ushio et al. (2004)

PGE2 can not only directly affect hepatocyte but also promote the proliferation and migration of liver cancer cells by affecting the tumor microenvironment (TME). Mannan-binding lectin (MBL) can activate the complement lectin pathway and prevent infection as part of TME. Moreover, Cox-2/PGE2/EP/VEGF pathway may also contribute to tumor angiogenesis in HCC (Zhao et al., 2007).

## Effect of PGE2 on the Tumor Microenvironment in Hepatocellular Carcinoma

The tumor microenvironment (TME) in hepatocellular carcinoma plays a critical role in hepatocarcinogenesis, liver fibrosis, tumor invasion, and metastasis. A series of studies have revealed the importance of TME components, such as tumor-associated macrophages (TAMs), hepatic stellate cells, and regulatory and cytotoxic T cells, in tumorigenesis and tumor progression (Mizuno et al., 2019). Here, we summarize the role of COX-2/PGE2 axis played in each component of HCC tumor microenvironment.

TAMs inhibit anti-tumor immunity and promote tumor progression by expressing cytokines and chemokines. High COX-2-expressing HCC cell lines can induce anti-tumor abilities’ exhaustion in activated CD8^+^ T cell through alternative (M2) tumor-associated macrophages polarization and TGF-β pathway. COX-2 inhibitors may reduce the inhibitory effect on CD8^+^ T cells through regulating TAMs in tumor immune microenvironment, thus enhancing the T cell-based cytotoxicity and improving the prognosis of HCC patients (Xun et al., 2021). Studies have found that ERK/COX-2/PGE2 signaling pathway is related to the activation of hepatic stellate cells (HSCs) (Li et al., 2019). And the HSCs-induced myeloid-derived suppressor cells (MDSC) accumulation and HCC growth is mediated by COX2-PGE2-EP4 pathway (Xu et al., 2016). In patients with advanced stage HBV-related liver fibrosis, researchers found that regulatory T cells and Th17 cells are upregulated by Hepatic stellate cells via PGE2-EP2 and PGE2-EP4 pathway (Li et al., 2017). In conclusion, PGE2 usually plays an anti-inflammation role on the tumor microenvironment in HCC to promote tumor invasion and metastasis.

## The Treatment Strategy Based on Cox-2/PGE2 Axis on Hepatocellular Carcinoma

As the Cox-2/PGE2 axis plays an important role in the progress of hepatocellular carcinoma, there are a number of experiments focusing on the treatment of HCC based on it. Due to the abundant effect of PGE2 in physiological and pathological progress, the single use of antagonist of EP receptor or abnormal dose inhibitor of synthesis enzyme for PGE2 is always accompanied with side effects. But combination use of drugs targeting Cox-2/PGE2 axis and traditional antitumor drugs show great potentials in HCC. T7 peptide is the N-terminal part of tumstatin, an endogenous angiogenic. Compared to tumstatin, T7 peptide has low molecular weight and shares similar activity in suppressing the proliferation, migration, and promotion of the apoptosis of endothelial cells. Combination use of selective Cox-2 inhibitor meloxicam and T7 peptide shows a greater anti-tumor effect against HCC tumor in mice (Yang et al., 2021). EP1 antagonist ONO-8711 enhanced the effect of EGCG, Epigallocatechin gallate, in inhibiting PGE2-induced HCC proliferation (Yang et al., 2019). The combination uses of drugs based on COX-2/PGE2 axis and traditional antitumor drugs show great potentials to diminish tumor progression of HCC *in vivo* and vitro, indicating that it might be a possible strategy for treatment of advanced primary hepatocellular carcinoma.

## Discussion

PGE2 is known as an important factor in inflammatory milieu that influence malignant tumor outset and progression. Researchers have found that PGE2 can promote various kinds of cancer cell growth by regulating immune response and enhancing resistance to apoptosis. Extensive clinical and epidemiological studies show that reduction of PGE2 level in tumor can rebuild tumor microenvironment by reprograming anti-tumor immunity, thus inhibiting tumor growth and metastasis. For example, a new study found that the selective COX-2 inhibitor celecoxib can be used *in vitro*, synergistically enhancing the inhibitory effect of sorafenib on cancer cell growth and AKT activation and inducing cancer cell apoptosis [70].

The clinical study of the anti-tumoral effect of PGE2 in cancer by using NSAIDs or COX-2 inhibitor has been performed with failed outcomes due to severe side effects (Mizuno et al., 2019). This suggests that drugs based on the COX-PGE2 axis may be used in combination with traditional anti-tumoral drugs to avoid serious side effects caused by high-dose medication alone. In HCC, evidence shows the pivotal role PGE2 played in the progression of tumor *in vitro*. But there are few clinical studies focusing of the COX-2/PGE2 axis in HCC. As more small-molecule ligands targeting EP receptors have been developed, therapy based on a combination of EP receptor and traditional anti-tumoral drugs is being considered.

In conclusion, PGE2 shows a traditional target with pleiotropic effects in tumorigenesis and progression of HCC to create a new potential clinical impact. For the treatment study focusing on the COX-PGE2 axis, the exclusive usage of NSAIDs or COX-2-inhibitors may be replaced by a combination of selective EP antagonists and traditional anti-tumoral drugs to alleviate severe side effects and achieve better outcomes.
